# Assessment of the Association of D2 Dopamine Receptor Gene and Reported Allele Frequencies With Alcohol Use Disorders: A Systematic Review and Meta-analysis

**DOI:** 10.1001/jamanetworkopen.2019.14940

**Published:** 2019-11-08

**Authors:** Yonwoo Jung, Rachel A. Montel, Pei-Hong Shen, Deborah C. Mash, David Goldman

**Affiliations:** 1 Laboratory of Neurogenetics, National Institute on Alcohol Abuse and Alcoholism, National Institutes of Health, Rockville, Maryland; 2 Department of Biological Sciences, Seton Hall University, South Orange, New Jersey; 3 Department of Neurology and Molecular and Cellular Pharmacology, University of Miami Miller School of Medicine, Miami, Florida; 4 Office of the Clinical Director, National Institute on Alcohol Abuse and Alcoholism, National Institutes of Health, Rockville, Maryland

## Abstract

**Importance:**

The association between the D2 dopamine receptor gene (*DRD2*) Taq1A locus (rs1800497) and alcohol use disorder (AUD) is enduring but the subject of long-standing controversy; meta-analysis of studies across 3 decades shows an association between rs1800497 and AUD, but genome-wide analyses have detected no role for rs1800497 in any phenotype. No evidence has emerged that rs1800497, which is located in *ANKK1*, perturbs the expression or function of *DRD2*.

**Objective:**

To resolve contradictions in previous studies by identifying hidden confounders and assaying for functional effects of rs1800497 and other loci in the *DRD2* region.

**Data Sources:**

PubMed (882 studies), Embase (1056 studies), and Web of Science (501 studies) databases were searched through August 2018. Three clinical populations—Finnish, Native American, and African American participants—were genotyped for 208 to 277 informative single-nucleotide polymorphisms (SNPs) across the *DRD2* region to test the associations of SNPs in this region with AUD.

**Study Selection:**

Eligible studies had diagnosis of AUD made by accepted criteria, reliable genotyping methods, sufficient genotype data to calculate odds ratios and 95% CIs, and availability of control allele frequencies or genotype frequencies.

**Data Extraction and Synthesis:**

After meta-analysis of 62 studies, metaregression was performed to detect between-study heterogeneity and to explore the effects of moderators, including deviations of cases and controls from allele frequencies in large population databases (ExAC and 1000 Genomes). Linkage to AUD and the effect on gene expression of rs1800497 were evaluated in the context of other SNPs in the *DRD2* region. Data analysis was performed from August 2018 to March 2019. This study follows the Preferred Reporting Items for Systematic Reviews and Meta-analyses reporting guideline.

**Main Outcomes and Measures:**

The effects of rs1800497 and other SNPs in the *DRD2* region on gene expression were measured in human postmortem brain samples via differential allelic expression and evaluated in other tissues via publicly available expression quantitative locus data.

**Results:**

A total of 62 studies of *DRD2* and AUD with 16 294 participants were meta-analyzed. The rs1800497 SNP was associated with AUD (odds ratio, 1.23; 95% CI, 1.14-1.31; *P* < .001). However, the association was attributable to spuriously low allele frequencies in controls in positive studies, which also accounted for some between-study heterogeneity (*I^2^* = 43%; 95% CI, 23%-58%; *Q*_61_ = 107.20). Differential allelic expression of human postmortem brain and analysis of expression quantitative loci in public data revealed that a cis-acting locus or loci perturb the *DRD2* transcript level; however, rs1800497 does not and is not in strong disequilibrium with such a locus. Across the *DRD2* region, other SNPs are more strongly associated with AUD than rs1800497, although no *DRD2* SNP was significantly associated in these 3 clinical samples.

**Conclusions and Relevance:**

In this meta-analysis, the significant association of *DRD2* with AUD was reassessed. The *DRD2* association was attributable to anomalously low control allele frequencies, not function, in positive studies. For genetic studies, statistical replication is not verification.

## Key Points

**Question:**

Is there a biological association between D2 dopamine receptor gene (*DRD2*) and alcohol use disorder?

**Findings:**

This meta-analysis of 62 studies including 16 294 participants found that the association between *DRD2* and alcohol and heterogeneity between studies are associated with spuriously low allele frequencies in positive studies rather than with any ability of the linked locus to drive transcription.

**Meaning:**

These observations regarding the factors behind the association between alcohol use disorder and *DRD2* and tactics to identify those factors may be relevant to other findings that are highly significant in meta-analyses but biologically meaningless and that may be associated with research and clinical care.

## Teaser

This meta-analysis examines the association between the D2 dopamine receptor gene (*DRD2*) and alcohol use disorder.

## Introduction

Whether the dopamine D2 receptor gene (*DRD2*) is associated with alcohol use disorder (AUD) and other behavioral phenotypes is a long-standing controversy. This discussion is driven by the role of 1 single-nucleotide polymorphism (SNP), rs1800497, which is located in a nearby gene, *ANKK1.* This SNP, from among hundreds now known in the *DRD2* region, was assayable as a restriction fragment–length polymorphism (RFLP) in 1990, when the association of *DRD2* and other genes with alcoholism was first examined.^1^ Newer technologies enabling large-scale genotyping of hundreds of SNPs in the *DRD2* region, and hundreds of thousands of SNPs genome-wide, have been applied in genome-wide association studies (GWAS) of many phenotypes, including AUD^2,3^ and related phenotypes,^4^ such as brain dopamine D2 binding potential.^5,6,7^ The disproportionate focus on rs1800497 has been amplified by positive meta-analyses, such that approximately 20 studies on the association between rs1800497 and AUD have been published per decade since 1990.

The advent of genomic technologies and the discovery that other loci in the *DRD2* region generate stronger, and even genome-wide significant, linkage signals^8^ has not diminished interest in rs1800497, which is marketed as a direct-to-consumer genetic test.^9^ In all GWAS in the GWAS catalog^10^ and UK BioBank database,^11^ no significant (or *P* < 10^−6^) (nominal) associations are reported between rs1800497 and any phenotype. However, in addition to the positive meta-analyses for rs1800497, other twists and turns have kept rs1800497 viable academically, as well as commercially. The initial report by Blum et al^1^ in 1990 was quickly followed by a negative study by Bolos et al^12^ in the same journal. That study and some subsequent negative studies^13^ were criticized on the basis of the idea that the controls might have had other phenotypes affected by the D2 dopamine receptor, and thereby might have been more likely to carry the rs1800497 T allele. Early on, although studies were still sparse, the possibilities that rs1800497 T allele frequencies were spuriously low and that the association might be attributable to population variation in allele frequencies were advanced by Gelernter et al,^14^ and consistent with this idea, studies conducted in well-defined populations, such as Finnish participants^14,15^ and Native American participants,^15^ were negative.

The association studies^1,16,17,18^ that drove interest in the rs1800497 locus and thereby the *DRD2* gene delivered very large effect sizes, with odds ratios (ORs) of greater than 3. This effect size is not out of line with that of the *ALDH2* Lys504^19^ allele and *ADH* gene cluster in AUD, as observed in GWAS,^20^ but is disproportionately large compared with the association with any locus ever implicated in GWAS of a psychiatric disease. Alcohol use disorder is clinically and etiologically heterogeneous, and genetic risk is strongly modulated by environmental interaction.^21^ In psychiatric disease GWAS, very large sample sizes (eg, >50 000 participants) are needed to identify genome-wide significant loci because these loci almost uniformly have ORs less than 1.1. One would not expect AUD to be an exception, except for gatekeeper polymorphisms, such as *ALDH2* Glu504Lys, which alters the metabolism of alcohol, and the Lys504 allele, which can lead to strong aversive effects. In contrast to very large psychiatric GWAS, all rs1800497 and AUD studies were conducted with fewer than 1000 participants, and collectively there were only 16 294 participants across 62 studies.^22,23,24,25,26,27,28,29,30,31,32,33,34,35,36,37,38,39,40,41,42,43,44,45,46,47,48,49,50,51,52,53,54,55,56,57,58,59,60,61,62,63,64,65,66,67,68,69,70,71,72,73,74,75,76,77,78,79^

We wished to identify potential causes of the association of rs1800497 with AUD observed in meta-analyses and to place the rs1800497 association with phenotype and gene expression in the context of other SNPs in the *DRD2* region. We meta-analyzed 62 studies but followed that analysis with metaregression to identify hidden confounders. Identification of the role of uncharacteristic rs1800497 allele frequencies was made possible by very large resources for population allele frequencies. To put rs1800497 in genomic context, we evaluated the association of SNPs in this region to AUD in 3 clinical populations, and for gene expression, we directly measured *DRD2* differential allele expression (DAE) in postmortem brain tissue samples, directly relating DAE to SNPs across the region encompassing *DRD2*. Furthermore, we exploited publicly available expression quantitative trait locus (eQTL) data to examine whether SNPs in the *DRD2* region estimated the expression of *DRD2* in other tissues where *DRD2* transcripts are measurable.

## Methods

### Study Search, Evaluation, and Selection

**Figure 1.**
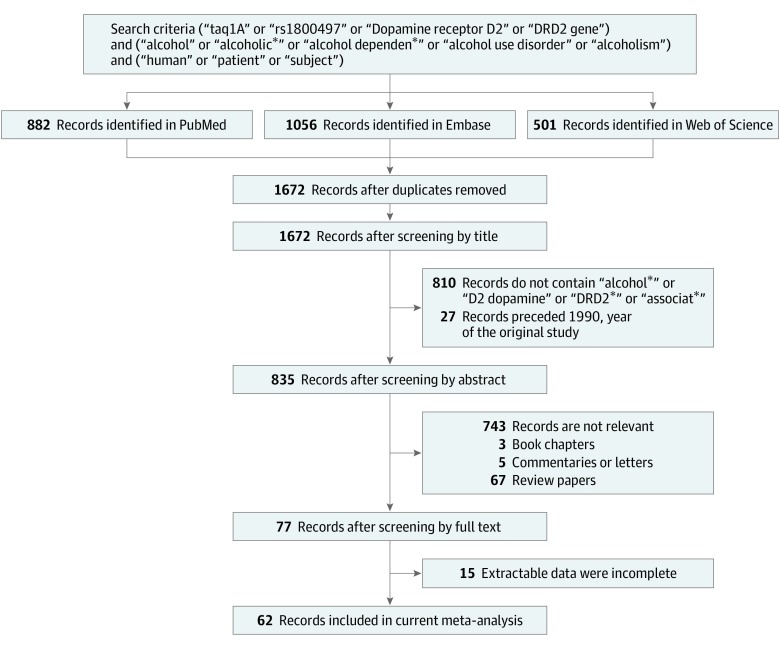
Workflow for Capture of Studies of Association Between D2 Dopamine Receptor Gene (*DRD2*) rs1800497 Single-Nucleotide Polymorphism and Alcohol Use Disorder In total, 882 records were identified in PubMed, 1056 records in Embase, and 501 records in Web of Science, with 77 records qualifying for meta-analysis after screening. Sixty-two records were used in the current meta-analysis after 15 studies were excluded because genotypes were incomplete or nonextractable.

Studies included in the meta-analysis were selected from PubMed, Embase, Web of Science, and Cochrane Library databases. The search was conducted through August 2018 using the following search logic: (Taq1A OR rs1800497 OR dopamine receptor D2 or *DRD2* gene) AND (alcohol OR alcoholic* OR alcohol dependen* OR alcohol use disorder OR alcoholism) AND (human OR patient OR subject). Duplicate studies were eliminated as shown in Figure 1. Previously published meta-analyses were examined to verify whether previously referenced studies about the association between *DRD2* and AUD had been detected.

The eligibility criteria were as follows: the diagnosis of AUD was made by use of accepted criteria, including *Diagnostic and Statistical Manual of Mental Disorders, Third Edition Revised* (*DSM-III-R*), *Diagnostic and Statistical Manual of Mental Disorders, Fourth Edition* (*DSM-IV*), *Diagnostic and Statistical Manual of Mental Disorders, Fifth Edition* (*DSM-5*), *International Statistical Classification of Diseases and Related Health Problems, Tenth Revision*, Diagnostic Interview for Genetic Studies, Michigan Alcohol Screening Test, and Feighner criteria; the genotyping methods included RFLP, 5′ exonuclease assay (TaqMan, Applied Biosystems), Sanger sequencing, array-based genotyping, or direct genotyping by any other reliable method; there were sufficient genotype data to calculate ORs and 95% CIs; and control allele frequencies or genotype frequencies were available. This study follows the Preferred Reporting Items for Systematic Reviews and Meta-analyses (PRISMA) reporting guideline.^17^

### Identification of Eligible Studies

We performed conventional meta-analysis in response to newer association studies published since that of Wang et al^24^ in 2013 and included or excluded studies on the basis of our criteria, which included requirements for certain genetic and procedural data. Our search strategy revealed a total of 882 publications from PubMed, 1506 from Web of Science, and 501 from Embase (Figure 1). All studies identified by Wang et al^24^ were captured by our search algorithm, although not all studies included in Wang et al met our criteria because those authors included studies for which allele frequencies could not be computed. References were imported into EndNote version X9.1 software (Thomson Reuters), and duplicates were removed. Additional studies were removed manually as described in Figure 1, and 62 studies were left eligible for the present meta-analysis. In addition to the 57 studies published before 2013 known to Wang et al,^24^ we included 5 studies published after 2013. Among these 62 studies,^1,6,12,15,16,17,18,25,26,27,28,29,30,31,32,33,34,35,36,37,38,39,40,41,42,43,44,45,46,47,48,49,50,51,52,53,54,55,56,57,58,59,60,61,62,63,64,65,66,67,68,69,70,71,72,73,74,75,76,77,78,79^ 24 analyzed the association between the rs1800497 T allele and AUD in Europe,^29,31,33,35,36,40,42,46,47,49,50,51,52,54,56,59,65,66,68,71,72,73,74,77^ 17 studies were from Asia,^30,34,37,38,41,43,44,53,55,61,63,64,67,69,70,75,76^ 15 were from North America,^1,6,12,15,16,17,18,25,26,27,28,32,39,58,62^ 3 were from South America,^48,56,78^ 2 were from Australia,^42,57^ and 1 was from Central America.^79^

### Data Extraction and Evaluation

Data were extracted from each study by authors and publication year, location of study, diagnostic criteria, numbers of cases and controls, genotype frequencies in cases and controls, and allele frequencies if available. Regions were classified as North America, South America, Europe, East Asia, South Asia, Africa, and Australia. Two researchers independently extracted data; disagreements would have been resolved in consensus, but there were none. Expected allele frequencies were based on population frequencies in the 1000 Genomes and ExAC databases. The χ^2^ distribution was used to test Hardy Weinberg equilibrium of genotypes. Data analysis was performed from August 2018 to March 2019.

### Genotyping

The association of 208 to 277 SNPs in the *DRD2* region with AUD was analyzed in 3 populations: 641 Finnish participants, 583 African American participants, and 501 Native American participants. All were studied following informed consent via protocols approved by the National Institutes of Health institutional review board, and all cases and controls were psychiatrically diagnosed using a structured interview. Additional details, including array-based genotyping methods, are in eAppendix 1 and eReferences in the Supplement.

### Differential Allelic Expression

Postmortem human cerebellum was obtained from the Miami Brain Bank (National Institute on Drug Abuse Brain Biorepository). For DAE, 28 brain samples heterozygous for rs62755, a reporter SNP in the *DRD2* transcript, were identified from a total of 82 brain samples screened by genotyping. Details on genotyping and DAE are in eAppendix 2 in the Supplement.

### Statistical Analysis

The association between rs1800497 and AUD was calculated from unadjusted ORs using a combination of contingency tables abstracted from each study. Pooled ORs and 95% CIs were calculated by a fixed-effect model (Mantel-Haenszel), random-effects model (restricted maximum likelihood), and mixed-effects model (general linear model). The effects of individual studies on pooled estimates were assessed by a sensitivity analysis. Subgroup analyses were performed to measure effects of location, diagnostic methods, and reported allele frequencies among controls and cases. Publication bias was assessed by the Begg rank correlation and Egger regression tests. All meta-analyses were performed using the Metafor package in R statistical software version 2.1-0 (R Project for Statistical Computing), Meta package version 4.9-6 (R Project for Statistical Computing), and Cochrane Review Manager version 5.3 statistical software (Cochrane Community). To measure effects of moderators, a mixed-effects model was used as described in the Metafor manual and other publications.^22,23^ This metaregression analysis sought to examine the contribution of moderators to true effect size. The association of *DRD2* rs1800497 with DAE of *DRD2* rs62755 reporter SNP alleles was tested using nonparametric rank-order statistics (Kruskal-Wallis, Mann-Whitney, and Levene tests). All statistical tests were 2-sided, and statistical significance was set at *P* < .05. To evaluate the association of *DRD2*-region SNPs with AUD, logistic regression was performed with European ancestry scores as covariates.

## Results

### Main Analysis and Subgroup Analyses

**Figure 2.**
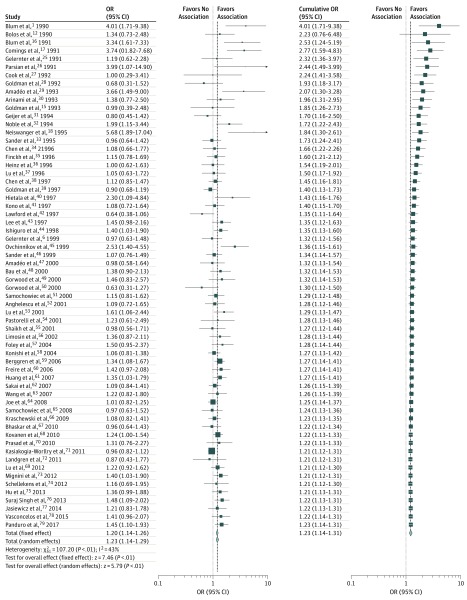
Meta-analysis of 62 Studies of the Association Between D2 Dopamine Receptor Gene rs1800497 Single-Nucleotide Polymorphism and Alcohol Use Disorder In the left panel, horizontal lines and squares represent 95% CIs and odds ratios (ORs) in each study. The estimated pooled effect size (represented by the different sizes of the squares) was calculated under fixed-effects and random-effects models. The cumulative plot (right panel) is sorted by publication year with pooled ORs (squares) calculated by adding each study sequentially. Diamonds denote total ORs, with their different sizes denoting different effect sizes.

The pooled OR estimates reveal that the rs1800497 T allele is associated with increased risk of alcohol dependence (OR, 1.23; 95% CI, 1.14-1.31; *P* < .001, random effects model) (Figure 2). However, moderately large heterogeneity was found across studies (*I^2^* = 43%; 95% CI, 23%-58%; *Q*_61_ = 107.20; *P* < .001), indicating that as much as one-fourth of the variance in AUD assignable to rs1800497 was attributable to heterogeneity. Subgroup analyses were performed to identify potential contributors to this heterogeneity, stratifying by study design, geographic location, method of diagnosis, and reported statistical significance. The rs1800497 T allele was associated with significantly elevated risk of alcohol dependence in all regions except Australia (Europe, OR, 1.16 [95% CI, 1.05-1.28]; North America, OR, 1.50 [95% CI, 1.15-1.95]; Asia, OR, 1.22 [95% CI, 1.12-1.33]; South America, OR, 1.40 [95% CI, 1.12-1.77]; and Central America, OR, 1.45 [95% CI, 1.10-1.93]) (eFigure 1 in the Supplement). Furthermore, the T allele was associated in studies with various diagnosis criteria (*DSM-III-R*, OR, 1.34 [95% CI, 1.16-1.55]; *DSM-IV*, OR, 1.21 [95% CI, 1.13-1.31]; *DSM-5*, OR, 1.45 [95% CI, 1.10-1.93]) (eFigure 2 in the Supplement). Although ORs of studies using older diagnostic criteria were higher, ORs were still significant in newer studies (eFigure 2 in the Supplement).

### Publication Bias

Association studies of *DRD2* were examined for publication bias, revealing an asymmetric funnel plot of log ORs (eFigure 3A in the Supplement). Although the Begg rank correlation test^80^ (τ = 0.141; *P* = .11) suggested less significant publication bias, the Egger regression test^81^ (*t* = 2.984; *df* = 60; *P* = .004) indicated bias, and the trim-and-fill method estimated that there were 6 missing publications (eFigure 3B in the Supplement). The strongest evidence of publication bias was in North American studies (*t* = 3.002; *df* = 14; *P* = .009, Egger test).

### Cumulative Analysis

Cumulative analysis (Figure 2) indicates a decrease in OR associated with rs1800497 over time. The high ORs observed in early studies, such as Blum et al^1^ (OR, 4.01; 95% CI, 1.71-9.38), were not observed in studies in later years, but the association with rs1800497 remained statistically significant. Furthermore, the cumulative OR of 1.23 (Cohen *d* = 0.68) (Figure 2) would represent a locus of large effect.

### Population Allele Frequency–Based Moderator Analyses and Metaregression

**Figure 3.**
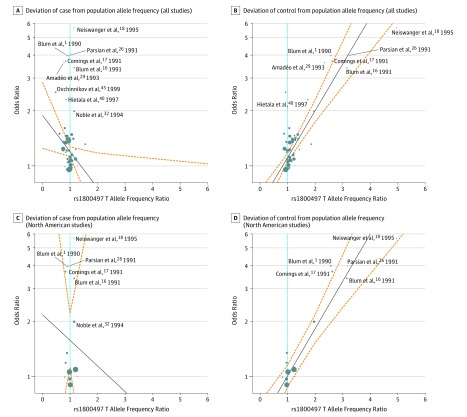
Metaregression With Case and Control rs1800497 Allele Frequency Ratios in All Studies and in North American Studies Only Metaregressions with case allele frequency ratio in all studies (*Z* = −2.11; *P* = .04) (A), control allele frequency ratio in all studies (*Z* = 7.73; *P* < .001) (B), case allele frequency ratio in North American studies (Z = −0.76; *P* = .44) (C), and control allele frequency ratio in North American studies (D) (*Z* = 6.09; *P* < .001) are shown. In each case, allele frequency is compared with population allele frequency in the 1000 Genomes database to detect allele frequency deviation. For comparison to ExAC database allele frequencies, see eFigure 4 in the Supplement. Diameters of circles are proportional to study population size. Solid lines represent the metaregression slopes of relationships of odds ratios to allele frequency deviation. Dashed lines denote 95% CIs. Allele frequency ratio is calculated by dividing population allele frequency with case or control allele frequency. In each graph, the cyan line indicates the point at which study allele frequency (rs1800497) is equal with population allele frequency.

Heterogeneity across all studies (*I*^2^ = 43%), and even higher heterogeneity in the North American studies (*I*^2^ = 71%), suggested the presence of a hidden confounding factor or factors (Figure 2; eFigure 1 in the Supplement). To identify this hidden moderator, we focused on diagnostic criteria and allele frequencies in cases and controls compared with population frequencies. Precedence for this latter analysis was set by Gelernter et al^14^ in 1993, who observed lower Taq1A (rs1800497 T) allele frequencies in controls in the few *DRD2* association studies in the literature at that time. The rs1800497 allele frequencies were evaluated for 57 studies; comparable population data or exact genotype numbers were unavailable in 5 studies^18,47,59,62,70^ for comparisons of genotypes expected and observed in cases and controls. In a metaregression analysis, aberrantly high ORs were observed to be associated with low T allele frequencies in controls (*Z* = 7.73; *P* < .001), and residual heterogeneity was reduced from 43% to 0.32%, regardless of whether 1000 Genomes (Figure 3B) or ExAC population allele frequency data (eFigure 4 in the Supplement) were used (*Z* = 7.76; *P* < .001). This finding also suggests that control allele frequency is the hidden variable behind the gradual decline in OR for the association with rs1800497, because several of the early studies were marked by very low T allele frequencies in controls. Interestingly, the allele frequency ratios comparing cases with population controls converge on 1 (Figure 3A and eFigure 4 in the Supplement). Large and statistically significant ORs reported in early studies such as Blum et al^1^ and Parsian et al^26^ correlate with significantly low T allele frequencies of the controls in these studies (Table and eTable in the Supplement). In studies in which rs1800497 was not associated with AUD, and when we examined the allele frequency in cases in studies overall, rs1800497 allele frequencies were consistent with population allele frequencies derived from the 1000 Genomes and ExAC databases.

**Table.** Deviations of Case and Control Allele Frequencies in D2 Dopamine Receptor Gene (rs1800497)/AUD Association Studies (1000 Allele Frequencies)^a^

Study	Total Alleles, No.	AUD Alleles, No.	Control Alleles, No.	Diagnostic Method	AUD		Control		Study OR (95% CI)	Ethnic Group Allele Frequency
					Allele Frequency Ratio	*P* Value	Allele Frequency Ratio	*P* Value		
Blum et al,^1^ 1990	140	70	70	*DSM-III-R*	0.89	.55	2.57	.003	4.01 (1.71-9.38)	0.28
Bolos et al,^12^ 1990	385	80	305	*DSM-III-R*	0.89	.64	0.97	.80	1.34 (0.73-2.48)	0.20
Blum et al,^16^ 1991	278	192	86	*DSM-III-R*	1.15	.29	3.15	<.001	3.43 (1.61-7.33)	0.28
Comings et al,^17^ 1991	346	208	138	*DSM-III-R*	0.84	.23	2.62	.002	3.74 (1.82-7.68)	0.20
Gelernter et al,^25^ 1991	224	88	136	*DSM-III-R*	0.84	.42	0.96	.82	1.19 (0.62-2.28)	0.20
Parsian et al,^26^ 1991	114	64	50	Feighner test	0.94	.81	3.17	.04	3.99 (1.07-14.90)	0.20
Cook et al,^27^ 1992	80	40	40	*DSM-III-R*	1.27	.56	1.27	.56	1.00 (0.29-3.41)	0.20
Goldman et al,^28^ 1992	164	92	72	*DSM-III-R*	1.12	.68	0.82	.43	0.68 (0.31-1.52)	0.23
Amadéo et al,^29^ 1993	184	98	86	*DSM-III-R*	0.78	.21	2.34	.02	3.66 (1.49-9.00)	0.20
Arinami et al,^30^ 1993	226	156	70	*DSM-III-R*	1.02	.90	1.25	.29	1.38 (0.77-2.50)	0.43
Goldman et al,^15^ 1993	92	44	48	Research Diagnostic Criteria	0.81	.23	0.81	.21	0.99 (1.15-3.44)	0.59
Geijer et al,^31^ 1994	310	148	162	*DSM-III-R*	1.08	.69	0.91	.57	0.80 (0.45-1.42)	0.20
Noble et al,^32^ 1994	306	146	160	*DSM-III-R*	1.15	.37	1.96	<.001	1.99 (1.15-3.44)	0.28
Neiswanger et al,^18^ 1995	164	104	60	*DSM-III-R*	NA	NA	NA	NA	5.68 (1.89-17.04)	0.20
Sander et al,^33^ 1995	766	540	226	*ICD-10*	1.04	.72	1.00	.99	0.96 (0.64-1.42)	0.20
Chen et al,^34^ 1996	398	316	82	*DSM-III-R*	1.00	.96	1.05	.78	1.08 (0.66-1.77)	0.43
Finckh et al,^35^ 1996	886	624	262	*ICD-10*	1.06	.55	1.19	.27	1.15 (0.78-1.69)	0.20
Heinz et al,^36^ 1996	420	194	226	*ICD-10*	1.00	.98	1.00	.99	1.00 (0.62-1.63)	0.20
Lu et al,^37^ 1996	252	122	130	*DSM-III-R*	0.91	.51	0.99	.92	1.05 (0.63-1.72)	0.43
Chen et al,^38^ 1997	832	406	426	*DSM-III-R*	1.00	.97	1.07	.38	1.12 (0.85-1.47)	0.43
Goldman et al,^39^ 1997	874	552	322	*DSM-III-R*	1.01	.86	0.97	.65	0.90 (0.68-1.19)	0.59
Hietala et al,^40^ 1997	240	140	100	*DSM-III-R*	0.86	.39	1.73	.07	2.30 (1.09-4.84)	0.23
Kono et al,^41^ 1997	386	200	186	*DSM-III-R*	1.05	.66	1.11	.41	1.08 (0.72-1.64)	0.43
Lawford et al,^42^ 1997	496	402	94	*DSM-III-R*	0.93	.52	0.66	.03	0.64 (0.38-1.06)	0.20
Lee et al,^43^ 1997	426	256	170	*DSM-III-R*	0.85	.08	1.06	.66	1.46 (0.98-2.16)	0.43
Ishiguro et al,^44^ 1998	722	418	304	*DSM-III-R*	0.96	.56	1.18	.10	1.40 (1.03-1.90)	0.43
Gelernter et al,^6^ 1999	592	320	272	*DSM-III-R*	0.99	.94	0.96	.80	0.97 (0.63-1.48)	0.23
Ovchinnikov et al,^45^ 1999	236	84	152	*DSM-III-R*	0.48	<.001	0.93	.69	2.53 (1.40-4.55)	0.20
Sander et al,^46^ 1999	1012	620	392	*DSM-III-R*	1.07	.46	1.13	.32	1.07 (0.76-1.49)	0.20
Amadéo et al,^47^ 2000	252	138	114	*DSM-III-R*	0.99	.92	0.97	.86	0.98 (0.58-1.64)	0.35
Bau et al,^48^ 2000	458	230	228	*DSM-III-R*	1.00	.99	1.28	.08	1.38 (0.90-2.13)	0.27
Gorwood et al,^49^ 2000	364	226	138	*DSM-III-R*	0.91	.54	1.25	.31	1.46 (0.83-2.57)	0.20
Gorwood et al,^50^ 2000	168	72	96	*DSM-III-R*	NA	NA	NA	NA	0.63 (0.31-1.27)	0.20
Samochowiec et al,^51^ 2000	968	584	384	Not stated	1.07	.51	1.20	.16	1.15 (0.81-1.62)	0.20
Anghelescu et al,^52^ 2001	682	486	196	*DSM-IV*	0.91	.37	0.98	.91	1.09 (0.72-1.65)	0.20
Lu et al,^53^ 2001	364	194	170	*DSM-III-R*	0.81	.04	1.06	.66	1.61 (1.06-2.44)	0.43
Pastorelli et al,^54^ 2001	248	120	128	*DSM-III-R*	1.20	.43	1.43	.14	1.23 (0.61-2.49)	0.20
Shaikh et al,^55^ 2001	206	100	106	Not stated	0.98	.05	0.97	.82	0.98 (0.56-1.71)	0.42
Limosin et al,^56^ 2002	454	240	214	DIGS	0.75	.02	0.95	.72	1.36 (0.87-2.11)	0.20
Foley et al,^57^ 2004	382	174	208	Not stated	0.62	.001	0.87	.36	1.50 (0.95-2.37)	0.20
Konishi et al,^58^ 2004	902	400	502	*DSM-IV*	0.98	.76	1.01	.90	1.06 (0.81-1.38)	0.49
Berggren et al,^59^ 2006	2398	714	1684	*DSM-IV*	0.85	.05	1.08	.17	1.34 (1.08-1.67)	0.20
Freire et al,^60^ 2006	664	200	464	*DSM-III-R*	0.98	.89	1.28	.02	1.42 (0.97-2.08)	0.27
Huang et al,^61^ 2007	854	452	402	*DSM-IV*	NA	NA	NA	NA	1.35 (1.03-1.79)	0.43
Sakai et al,^62^ 2007	1252	478	199	*DSM-IV*	1.20	.03	1.23	.005	1.09 (0.84-1.41)	0.28
Wang et al,^63^ 2007	462	146	316	*DSM-IV*	0.88	.29	0.98	.83	1.22 (0.82-1.80)	0.43
Joe et al,^64^ 2008	1604	1058	546	*DSM-IV*	NA	NA	NA	NA	1.01 (0.82-1.25)	0.43
Samochowiec et al,^65^ 2008	544	244	300	*DSM-IV*	1.06	.62	1.06	.68	0.97 (0.63-1.52)	0.20
Kraschewski et al,^66^ 2009	1456	720	736	*ICD-10*	1.02	.81	1.09	.35	1.08 (0.82-1.41)	0.20
Bhaskar et al,^67^ 2010	392	162	230	MAST	0.89	.29	0.87	.13	0.96 (0.64-1.43)	0.43
Kovanen et al,^68^ 2010	2046	1024	1022	*DSM-IV*	0.75	<.001	0.89	.09	1.24 (1.00-1.54)	0.23
Prasad et al,^70^ 2010	300	180	240	*DSM-IV*	1.55	.002	1.86	.001	1.31 (0.76-2.27)	0.43
Kasiakogia-Worlley et al,^71^ 2011	3922	2012	1910	Not stated	NA	NA	NA	NA	0.96 (0.82-1.12)	0.43
Landgren et al,^72^ 2011	232	168	64	*DSM-IV*	0.97	.85	0.87	.60	0.87 (0.43-1.77)	0.43
Lu et al,^69^ 2012	1082	266	816	*DSM-IV*	0.95	.57	1.07	.24	1.22 (0.92-1.62)	0.20
Mignini et al,^73^ 2012	1096	550	546	*DSM-IV*	0.89	.19	1.17	.14	1.40 (1.03-1.90)	0.20
Schellekens et al,^74^ 2012	418	220	198	*DSM-IV*	1.07	.67	1.22	.28	1.16 (0.69-1.95)	0.20
Hu et al,^75^ 2013	858	202	656	*DSM-IV*	0.97	.81	1.18	.01	1.36 (0.99-1.88)	0.43
Suraj Singh et al,^76^ 2013	830	258	572	*DSM-IV*	1.06	.03	1.37	<.001	1.48 (1.09-2.02)	0.43
Jasiewicz et al,^77^ 2014	652	338	314	*ICD-10*	0.87	.22	1.01	.92	1.21 (0.83-1.78)	0.20
Vasconcelos et al,^78^ 2015	454	226	228	*DSM-IV*	1.12	.23	1.39	.005	1.41 (0.96-2.07)	0.49
Panduro et al,^79^ 2017	908	624	284	*DSM-V*	0.89	.04	1.08	.38	1.45 (1.10-1.93)	0.49

^a^ Abbreviations: AUD, alcohol use disorder; DIGS, Diagnostic Interview for Genetic Studies; *DSM-III-R*,* Diagnostic and Statistical Manual of Mental Disorders (Third Edition Revised)*; *DSM-IV*,* Diagnostic and Statistical Manual of Mental Disorders (Fourth Edition)*; *ICD-10*,* International Statistical Classification of Diseases and Related Health Problems, Tenth Revision*; MAST, Michigan Alcohol Screening Test; NA, not applicable; OR, odds ratio.

To better understand the association between uncharacteristically low control allele frequencies and large ORs observed in some *DRD2* association studies and to characterize an overall, meta-analytically significant association, we grouped the studies as highly significant (OR, 1.64; 95% CI, 1.25-2.14), moderately significant (OR, 1.24; 95% CI, 1.15-1.32), or nonsignificant (OR, 1.09; 95% CI, 0.96-1.23). Next, within each category, we ranked studies from top to bottom according to how aberrant the control allele frequency was (eFigure 5 in the Supplement). Ten of 13 highly significant studies showed statistically significant deviation in control allele frequency. None of the 44 other studies did (χ^2^ = 15.14; *P* < .001).

### Other SNPs in the *DRD2* Region

**Figure 4.**
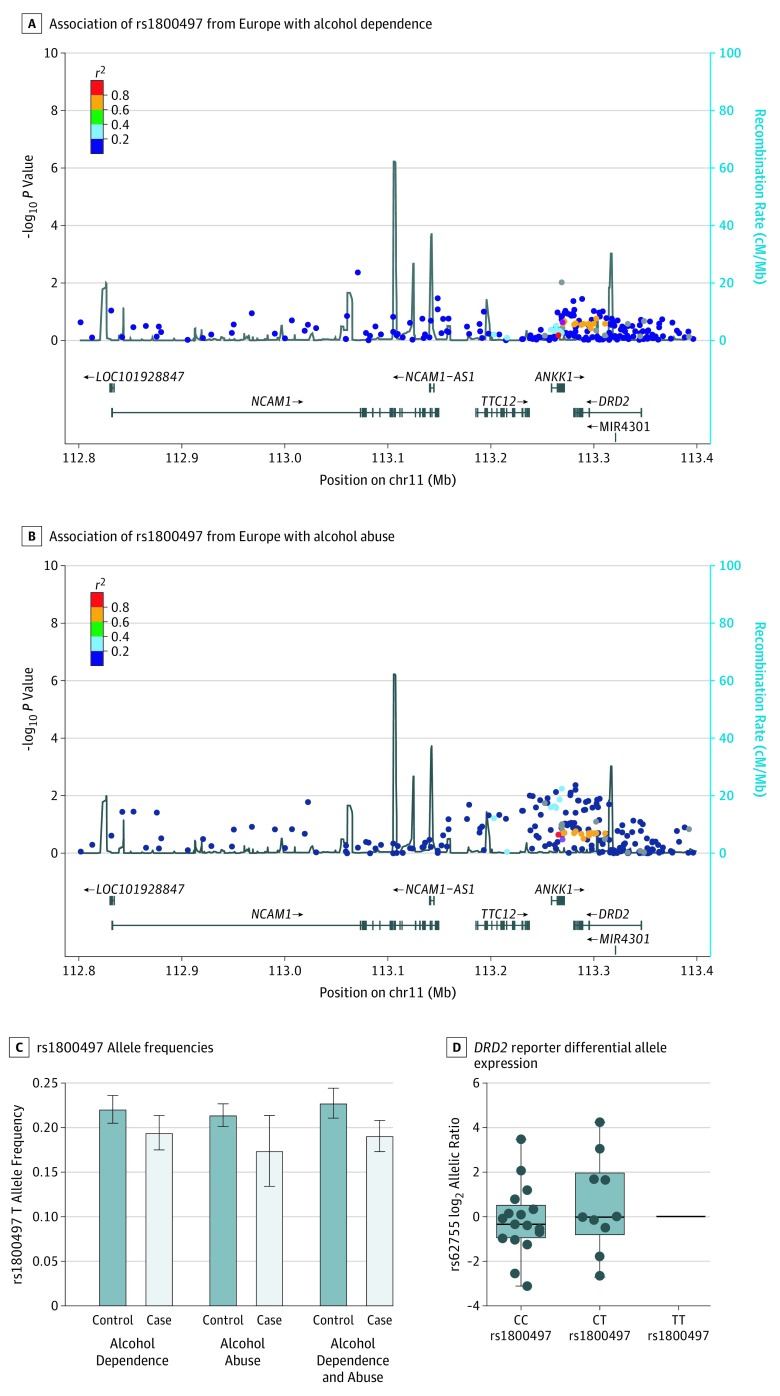
Linkage and Function in the D2 Dopamine Receptor Gene (*DRD2*) Region A and B, Regional association plots of rs1800497 and a total of 220 SNPs in the *DRD2* region are shown for alcohol dependence (A) and alcohol abuse (B) among 641 Finnish participants. Parallel local association plots are shown for 501 Native American and 583 African American participants in eFigures 6 and 7 in the Supplement. Single-nucleotide polymorphisms (dots) are color coded according to linkage disequilibrium (LD) with rs1800497 (red dot) on a scale of *r*^2^ 0 to 1. Estimated recombination rates (lines) reflect local LD structure in the 600 kb buffer around rs1800497 (red dot) in the Finnish population. C, rs1800497 allele frequencies did not differ between cases and controls. Vertical lines and whiskers denote 95% CIs. D, Differential allelic expression (DAE) of *DRD2* detected as deviation from 1:1 ratio of the alleles at a reporter locus (rs62755), indicating a cis-acting locus differentially driving *DRD2* transcript expression in 28 human postmortem brains heterozygous for the reporter locus, and identified from a larger number of brains. As shown, rs1800497 genotype is not associated with *DRD2* DAE (Kruskal-Wallis test, χ^2^_3_ = 0.7579; *P* = .68; Levene test, *F* = 2,25; *P* = .38). Top and bottom of boxes are 25th and 75th percentiles, respectively, lines inside boxes are medians, vertical lines are 10th and 90th percentiles, respectively, and circles denote individual data points. C indicates cytosine; and T, thymine.

With regard to genotyping arrays commonly used in genetic association analyses, such as the Infinium array with Exome content that we used (Illumina), many SNPs in the *DRD2* region and neighboring rs1800497 have been genotyped, including 220 on the Infinium array in the 600 kb region encompassing *DRD2*, *ANKK1* where rs1800497 is located, and other nearby genes (Figure 4A and B). If the association between rs1800497 and AUD were biologically valid, it would reflect the action of one of these SNPs or a nearby functional locus with which rs1800497 is in linkage disequilibrium (LD) (heat mapped in Figure 4 and eFigure 6 and eFigure 7 in the Supplement, showing that LD varies somewhat between populations). However, the Manhattan plots of the *DRD2* region for AUD in 3 populations reveal that rs1800497 is not associated with AUD, whereas other SNPs in the region generate stronger, albeit not genome-wide significant, signals of association (Figure 4 and eFigure 6 and eFigure 7 in the Supplement).

### DAE Analysis

We measured *DRD2* DAE in human brain tissue samples to determine whether rs1800497 or any other locus in the region drove *DRD2* expression. The reporter SNP in the *DRD2* transcript was selected as described in the Methods section, and only samples that were heterozygous for the reporter SNP were analyzed for DAE. Notably, DAE provides strong evidence for a cis-acting locus, or loci, driving variation in expression of the *DRD2* transcript. In postmortem hippocampus, 10 of 20 samples showed evidence of at least a 2-fold difference in *DRD2* transcript driven by a cis-acting locus. However, we found no significant association of rs1800497 with *DRD2* expression (Figure 4D).

## Discussion

This meta-analysis of 62 studies confirms the association of rs1800497 with AUD, as has been observed previously. The genotype-attributable OR for rs1800497 is 1.23, which would make rs1800497 one of the loci of largest effect ever observed for a common polymorphism on a behavioral phenotype. In large cohort addictions GWAS,^19,82^ the 2 genes of largest effect are *CHRNA5*, with an OR for smoking of 1.91 (95% CI, 1.01-11.99),^82^ and *ADH1B*, with an OR of 1.06 (95% CI, 0.94-1.19) for smoking and an OR of 1.02 (95% CI, 0.90-1.15) for alcohol.^19^ Furthermore, in a very large, meta-analytic nicotine GWAS,^83^ *DRD2* was a marginally significant gene, but rs1800497 was not implicated.

Another indicator that the association between rs1800497 and AUD does not have a functional origin is that haplotype-based studies^5,6^ conducted more than a decade ago implicated *DRD2*, but again the rs1800497 locus was not part of the haplotypes involved. Family-based studies, including association via the transmission disequilibrium test, which are less prone to ethnic stratification bias, do not support an association between rs1800497 and AUD.^39,84,85,86,87^ In a genomic context, rs1800497 is 1 of more than 20 million human SNPs, 1 of more than 1000 SNPs, and 1 of a much larger number of single-nucleotide variants in *DRD2* and genes as near to *DRD2* as *ANKK1*, where rs1800497 is located. As shown in Figure 4A and B, where multiple nearby SNPs in the *DRD2* region generate similar genetic association signals, many SNPs in the *DRD2 *and *ANKK1* region are in strong LD. Haplotype-based analyses can reduce the problem of multiple testing of SNPs that are genetically nonindependent and can also help focus on the functional locus, which is a virtue of allele-based linkage performed in association studies.

Here, we performed association analysis against AUD in 3 populations using 208 to 277 array-genotyped SNPs spanning the 600 kb region encompassing *DRD2* and flanking genes. Samples of the sizes we used (eg, 641 Finnish participants) are insufficient to detect loci with ORs much less than 1.1, as may be detectable in very large GWAS. However, analyzing only the local *DRD2* gene region, each sample could detect an OR of 1.23 (Cohen *d* = 0.68) and would be powered genome-wide for ORs much greater than 2, as claimed in many of the positive reports shown in Figure 2. For example, χ^2^ values greater than 35 would be expected for ORs greater than 2 in samples of this size. As discussed, rs1800497 is not represented in the GWAS catalog, having never been linked to any phenotype via GWAS. Notably, rs1800497 was not implicated in large GWAS of AUD and alcohol drinking.^11,88^ Other SNPs in the *DRD2* region that have been linked to phenotypes such as smoking have been identified in very large case-control data sets, and their effect sizes are small (OR, <1.1).^19^ Here we have observed that in the *DRD2* region, no SNP is significantly linked to AUD but the strongest signals of nominal association are to other SNPs, and these SNPs are not in LD with rs1800497. The local association plot (Figure 4A and B and eFigure 1 and eFigure 2 in the Supplement) showing these association signals emphasizes that rs1800497 is 1 SNP of many in the *DRD2* region.

In recent years, *DRD2* association studies have largely returned, or regressed, from analyses at the multilocus and haplotype levels to analyses of the single SNP, rs1800497. Justifications include replication of previous results and studies of rs1800497 in other contexts, or against other measures, and without subtracting power via multiple testing. However, the continued focus on rs1800497 has impeded understanding of the gene, much as if studies of sickle cell anemia had not advanced from use of the Hpa1 RFLP, discovered by Kan et al^89^ in 1978, to the *HBB* Val6 missense variant that causes sickle cell anemia and with which the RFLP discovered by Kan et al is in LD. If rs1800497 altered expression of *DRD2* transcript or function of the receptor, it would be logical to directly genotype it as the functional locus, rather than genotyping the proxy loci. However, rs1800497 is not a functional SNP but a legacy genetic marker, having been analyzed in the late 1980s as a Taq1A RFLP on Southern blots.^90^ The Taq1A restriction site is not located in *DRD2* but resides in a nearby gene, *ANKK1*. Later, and reflecting its somewhat high numerical designation, the *ANKK1* Taq1A polymorphism was designated rs1800497.

In this study, we directly tested whether rs1800497 is functional via its capacity to drive DAE of *DRD2* and by searching publicly available eQTL data for association with *DRD2* expression in various tissues. Intriguingly, the DAE analysis, controlling for trans-acting factors, provides strong evidence for the existence of a cis-acting locus or loci that alters the expression of *DRD2*. Differential expression of reporter alleles in heterozygotes is not correlated with trans-acting factors but with some genetic element acting in cis on the same chromosome.^91^ In postmortem hippocampus, 10 of 20 samples showed evidence of at least a 2-fold difference in *DRD2* transcript driven by a cis-acting locus. However, in the data we generated and data that are publicly available in the GTEx database, rs1800497 is not a cis-eQTL for *DRD2* or any nearby gene.

Heterogeneity analysis can indicate the presence of hidden confounders that can both drive and obscure associations. Therefore, when heterogeneity is detected, isolation of the source may clarify and enhance a true biological association. For the association between *DRD2* and AUD, moderately large heterogeneity was observed (*I^2^* = 43%), indicating that as much as one-fourth of the variance in AUD assignable to rs1800497 was attributable to heterogeneity. Heterogeneity was highest in North American studies (*I^2^* = 71%), indicating that it might be particularly beneficial to search for confounders in those studies. Under some circumstances, metaregression analysis can identify a confounder, but it is usually necessary to target variables that could alter the result. Using allele frequency data now available in large, publicly accessible databases, we were able to show that approximately 43% of the heterogeneity-attributable variance can be assigned to anomalously low control allele frequencies. Low rs1800497 T allele frequencies in controls are greatly overrepresented in positive studies, particularly in earlier studies in which the highest ORs were observed. Other potential sources of the remaining heterogeneity, under the premise that the rs1800497 association is biologically meaningful, include that gene and environment interactions vary across place and time and that populations differ in LD or the frequency of whatever functional allele to which rs1800497 might be linked.

It is interesting that the possibility that low control allele frequencies drove *DRD2* associations was noted relatively early by Gelernter et al.^14^ However, at that time, few large-scale resources for population allele frequencies were available, and the record of *DRD2* association, with each study representing a data point for meta-analysis, was sparse. After the publication by Gelernter et al,^14^ the explanation that population stratification drove strong *DRD2* associations was widely discounted, or ignored, not being mentioned in positive *DRD2* association papers published from 1993 to the present, or in meta-analyses that continued to confirm the association of *DRD2* with AUD.^24^

The counterpoint to the anomalously low allele frequencies in controls is allele frequency in cases. Here, we were interested to see that the case allele frequency did not, on an overall basis, drive the association between *DRD2* and AUD one way or another. Across all studies, rs1800497 T allele frequency in cases is similar to that in population controls, and in most individual studies, the ratio of case to population control allele frequency is approximately 1:1. Occasionally, it has been argued that it is essential to identify, and to remove, cases from population controls. Doing so can, of course, accentuate a case-control OR.

Some publication bias for positive *DRD2* association is observable. This bias in publication is insufficient to drive the association to the overall meta-analytic OR of 1.23. The studies analyzed here, which were conducted in several regions of the world (Asia, Europe, and North America), have reported high ORs. However, on an overall basis, the strongest evidence of publication bias is in North America studies, including early studies reporting very high OR that sparked the strongest interest in rs1800497 and the harshest debates. Here, we have shown that North American studies with anomalously low control allele frequencies can account for the publication bias in studies from that region.

### Limitations

Although we have now confirmed that low control allele frequencies drove the meta-analytically significant associations between *DRD2* and AUD, there is still an unresolved issue of why in the studies with large ORs the frequencies of rs1800497 T allele are generally lower in the controls. This is a limitation of our study. Ethnic stratification can occur whenever there is a systematic ancestral difference in allele frequency between cases and controls. If not taken into account, population stratification can lead to false-positive or false-negative results. By using ancestry principal components, GWAS can detect and at least partly correct for ethnic stratification, and, as noted, rs1800497 was not detected in GWAS of addictions. Notably, rs1800497 is an ancestry-informative locus, with a T allele frequency as high as 0.83 in some Native American tribes.^92^ The T allele frequency is as low as 0.08 to 0.11 in Ashkenazi and Yemenite Jewish populations and is also low in several other populations for which sufficient numbers have been genotyped.^92^ Most populations have higher, but still highly variable, T allele frequencies where very large numbers of population controls have been genotyped. In the ExAC database as of August 2019, the T allele frequencies were 0.20 in European, 0.30 in South Asian, 0.37 in African, and 0.49 in Latino populations. Speculatively, because ancestry was not reported and seldom was measured in single-locus *DRD2* association studies, some of these studies may have been stratified by ancestry, and, for example, may have included more controls of Jewish ancestry. However, in the absence of detailed information on ethnic origins or ancestry informative markers, this remains speculative.

The evidence from DAE that a cis-acting locus or loci drives *DRD2* expression can encourage and inform studies associating *DRD2* with phenotypic variation. The dopamine D2 receptor is integral to many behaviors, including addictions. The integration of genotype and haplotype information with functional variation, identification of functional loci using gene-specific data such as those generated here, and the use of new tools embodied in initiatives such as Encode^93^ and PsychEncode^94^ can inform and accelerate understanding of the association of *DRD2* with behavior.

## Conclusions

The *DRD2* gene (specifically, the SNP rs1800497) remains meta-analytically associated with AUD, with a high OR of 1.23, but the association is attributable to anomalously low control allele frequencies in studies driving the association. Placing the rs18000497 locus in context, we evaluated linkage to AUD using many SNPs in the *DRD2* region, and in the context of published GWAS, none of these data implicated rs1800497. Critical to future genetic studies on *DRD2* is the presence of cis-acting loci altering expression of this gene, as evidenced by both differential expression of alleles at a reporter locus in brain and publicly available cis-eQTL data. Beyond rs1800497, genomic analyses unbiased by the legacy of which marker happened to be genotyped first can focus on loci associated with the function of *DRD2* that modulate the numerous phenotypes that are, in turn, modulated by the dopamine D2 receptor.

## Notes

Supplement. **eAppendix 1. **Sample, Diagnosis, and Consent Information **eReferences.** **eAppendix 2.** Methods **eFigure 1.** Meta-analysis of *DRD2* rs1800497/AUD Studies Stratified by Region **eFigure 2.** Meta-analysis of *DRD2* rs1800497/AUD Studies Stratified by Diagnostic Criteria **eFigure 3.** Publication Bias **eFigure 4.** Metaregression Plots **eFigure 5.** Meta-analysis of rs1800497 Association With AUD Stratified by Deviation Significance **eFigure 6.** Association of SNPs in the *DRD2* Region (Native Americans) **eFigure 7.** Association of SNPs in the *DRD2* Region (African Americans) **eTable.** Deviations of Case and Control Allele Frequencies in *DRD2* (rs1800497)/AUD Association Studies (ExAC Allele Frequencies)
