# Supplementary material for: Assessment of the Association of D2 Dopamine Receptor Gene and Reported Allele Frequencies With Alcohol Use Disorders: A Systematic Review and Meta-analysis
Source: JAMA Netw Open. 2019 Nov 8;2(11):e1914940. doi: 10.1001/jamanetworkopen.2019.14940 (PMC6902783; doi:10.1001/jamanetworkopen.2019.14940)
Supplement: Supplement. — eAppendix 1. Sample, Diagnosis, and Consent Information eReferences. eAppendix 2. Methods eFigure 1. Meta-analysis of DRD2 rs1800497/AUD Studies Stratified by Region eFigure 2. Meta-analysis of DRD2 rs1800497/AUD Studies Stratified by Diagnostic Criteria eFigure 3. Publication Bias eFigure 4. Metaregression Plots eFigure 5. Meta-analysis of rs1800497 Association With AUD Stratified by Deviation Significance eFigure 6. Association of SNPs in the DRD2 Region (Native Americans) eFigure 7. Association of SNPs in the DRD2 Region (African Americans) eTable. Deviations of Case and Control Allele Frequencies in DRD2 (rs1800497)/AUD Association Studies (ExAC Allele Frequencies) [file jamanetwopen-2-e1914940-s001.pdf]

Supplementary Online Content

Jung Y, Montel RA, Shen P-H, Mash DC, Goldman D. Assessment of the association of D2dopamine receptor gene and reported allele frequencies with alcohol use disorders: a systematic review and meta-analysis. *JAMA Netw Open*. 2019;2(11):e1914940. doi:10.1001/jamanetworkopen.2019.14940

eAppendix 1. Sample, Diagnosis, and Consent Information

eReferences

eAppendix 2. Methods

eFigure 1. Meta-analysis of *DRD2* rs1800497/AUD Studies Stratified by Region

eFigure 2. Meta-analysis of *DRD2* rs1800497/AUD Studies Stratified by Diagnostic Criteria

eFigure 3. Publication Bias

eFigure 4. Meta-Regression Plots

eFigure 5. Meta-analysis of rs1800497 Association With AUD Stratified by Deviation Significance

eFigure 6. Association of SNPs in the *DRD2* Region (Native Americans)

eFigure 7. Association of SNPs in the *DRD2* Region (African Americans)

eTable. Deviations of Case and Control Allele Frequencies in *DRD2* (rs1800497)/AUD Association Studies (ExAC Allele Frequencies)

This supplementary material has been provided by the authors to give readers additional information about their work.

eAppendix 1. Sample, Diagnosis, and Consent Information

General description

- All the SNPs were genotyped using smokescreen array (https://bmcgenomics.biomedcentral.com/articles/10.1186/s12864-016-2495-7). SNPs with MAF>0.005 were included in the plots.

Finnish Caucasians

- The sample from Helsinki, Finland, has been described in detail elsewhere (GABRG1 and GABRA2 as independent predictors for alcoholism in two populations, Enoch et al).[1] All participants gave written informed consent to the study that was approved by the IRBs of the National Institute on Alcohol Abuse and Alcoholism (NIAAA) and the National Institute of Mental Health (NIMH), the University of Helsinki, and the University of Helsinki Central Hospital, Helsinki, Finland.
- Amount 641 genotyped subjects in the plots, 533 were males and 108 were females.
- There are 55 Alcohol Abuse cases and 586 controls. There are 289 Alcohol Dependent or Abuse cases, 352 controls. There are 234 Alcohol Dependent cases, 407 controls. Psychiatric diagnoses were based on DSM-III.
- Logistic regression was performed with European AIMs scores as covariates. (Using ancestry-informative markers to define populations and detect population stratification. Enoch et al).[1]

African American

- African-American patients were recruited from the Substance Abuse Treatment Program (SATP) at the Department of Veteran Affairs New Jersey Healthcare System (VANJHCS), East Orange Campus. A detailed description of this sample has previously been provided

© 2019 Jung Y et al. *JAMA Network Open.*

(Two HPA axis genes, CRHBP and FKBP5, interact with childhood trauma to increase the risk for suicidal behavior. Roy, 2012). All participants gave written informed consent to the study that was approved by the Institutional Review Boards (IRBs) of the Department of Veteran Affairs New Jersey Healthcare System (VANJHCS) and the University of Medicine and Dentistry, New Jersey Medical School (UMDNJ).

- Amount 583 genotyped subjects in the plots, 356 were males and 227 were females.
- There are 163 Cocaine dependent cases and 420 controls. There are 119 Heroine Dependent cases, 464 controls. There are 161 Alcohol Dependent cases, 422 controls. Psychiatric diagnoses were based on DSM-IV.
- Logistic regression was performed with Africa AIMs scores as covariates. (Using ancestry-informative markers to define populations and detect population stratification. Enoch et al).[1]

American Indian

- From Southwestern American Indian tribe. All subjects gave informed consent under a human research protocol approved by the Institutional Review Board of the National Institute on Alcohol Abuse and Alcoholism and the Tribal Council. Most participants derived from three large interrelated pedigrees; however, the average sharing of descent between any two individuals was 0.012 which is equivalent to the relationship between second cousins once removed and third cousins. For a more detailed description see Relationship of binge drinking to alcohol dependence, other psychiatric disorders, and behavioral problems in an American Indian tribe. Robin et al).[2]
- Amount 501 genotyped subjects in the plots, 217 were males and 283 were females.

© 2019 Jung Y et al. *JAMA Network Open.*

- There are 30 Alcohol Abuse cases and 471 controls. There are 353 Alcohol Dependent or Abuse cases, 148 controls. There are 323 Alcohol Dependent cases, 178 controls. Psychiatric diagnoses were based on DSM-III.
- Logistic regression was performed with American Indian AIMs scores as covariates. (Using ancestry-informative markers to define populations and detect population stratification. Enoch et al).[1]

eReferences

1. Enoch M-A, Shen P-H, Xu K, Hodgkinson C, Goldman D. Using ancestry-informative markers to define populations and detect population stratification. *J Psychopharmacol Oxf Engl*. 2006;20(4 Suppl):19-26. doi:10.1177/1359786806066041

2. Robin RW, Long JC, Rasmussen JK, Albaugh B, Goldman D. Relationship of binge drinking to alcohol dependence, other psychiatric disorders, and behavioral problems in an American Indian tribe. *Alcohol Clin Exp Res*. 1998;22(2):518-5236.

eAppendix 2. Methods

Array based genotyping

Array-based SNP genotyping was performed using Illumina GoldenGate protocols on 96-well format BioRealm Smokescreen arrays. Five hundred nanograms of genomic DNA was used per assay. Pre-PCR DNA processing was performed using a TECAN liquid handling robot running Illumina protocols. Arrays were imaged using an Illumina Beadstation GX500 and data analyzed using GenCall v6.2.0.4 and GTS Reports software v5.1.2.0 (Illumina). Genotype clusters were determined for a test dataset and this template was applied to all subsequent datasets. Data were polished by manual adjustment of the clustering for each SNP to correct for differences between datasets arising from sample integrity and concentration. The GenCall score is a value between 0

© 2019 Jung Y et al. *JAMA Network Open.*

and 1 giving a confidence score for that genotype call (the higher the score the higher the confidence in the call) and is derived from the tightness of the clusters for a given locus and the position of the sample relative to its cluster. Loci with call rate <90% were determined to have failed and were excluded. At this point deviation from Hardy–Weinberg equilibrium was not used as an exclusion criterion because all datasets contained both case and control samples, potentially driving deviation from HWE.

Differential allelic expression (DAE) and SNP genotyping in human brain

Postmortem brain tissue was provided by the University of Miami Brain Bank. Specimens were obtained at autopsy from chronic cocaine and alcohol abusers and age-matched drug- and alcohol-free control subjects. All subjects died suddenly without a prolonged agonal state. Brain pH measures were done as quality control for each case with values > 6.0. Postmortem samples were genotyped for rs1800497 and rs62755, the latter being the reporter locus for DAE. These SNPs were PCR-amplified and genotyped in all subjects using custom 5' exonuclease primers and probes (Life Technologies). Genotypes were resolved by size using an ABI 3730 Capillary Sequencer and GeneMapper Software v4.0.

***DRD2* differential allelic expression**

To determine if there is a cis-acting eQTL affecting expression of *DRD2*, and if rs1800497 is a cis-acting eQTL, the expression of alleles for the reporter SNP rs62755 was compared in 28 heterozygous brain samples identified from a larger (N=82) number of brains. The reporter SNP was selected on the basis that it was exonic with an allele frequency approaching 0.5, thereby ensuring that multiple heterozygous samples would be available for analysis. The messenger RNA levels for both alleles were simultaneously quantified by real-time PCR on hippocampal

© 2019 Jung Y et al. *JAMA Network Open.*

RNA using a Custom Taqman SNP Genotyping Assay (Life Technologies). The complementary DNA (cDNA) was quantified on a QuantStudio 7 Flex Real-Time PCR System (Life Technologies) in a 10 µL qRT-PCR reaction: 2 µL cDNA, 5 µL Amplitaq Gold 360 PCR MasterMix, 0.25 µL custom primer/probe assay, 2.75 µL water. The reaction conditions were as follows: 10 min at 95 °C, and 45 cycles of: 15 s at 95 °C and 1 min at 60 °C. Each sample was analyzed in triplicate. The average difference in Ct (threshold cycle) between the two alleles for cDNA samples was normalized against the average difference in Ct between the two alleles in genomic DNA samples.

Study or Subgroup	Experimental Events	Experimental Total	Control Events	Control Total	Weight (fixed)	Weight (random)	Odds Ratio MH, Fixed + Random, 95% CI
**region = North America**							
Blum 1990	26	70	9	70	0.2%	0.6%	4.01 [1.71, 9.38]
Bolos 1990	17	80	51	305	0.5%	1.0%	1.34 [0.73, 2.48]
Blum 1991	55	192	9	86	0.3%	0.7%	3.43 [1.61, 7.33]
Comings 1991	47	208	10	138	0.3%	0.8%	3.74 [1.82, 7.68]
Gelernter 1991	20	88	27	136	0.5%	0.9%	1.19 [0.62, 2.28]
Parsian 1991	13	64	3	50	0.1%	0.3%	3.99 [1.07, 14.90]
Cook 1992	6	40	6	40	0.2%	0.3%	1.00 [0.29, 3.41]
Goldman 1992	14	92	15	72	0.5%	0.6%	0.68 [0.31, 1.52]
Goldman 1993	32	44	35	48	0.3%	0.5%	0.99 [0.39, 2.48]
Noble 1994	42	146	27	160	0.6%	1.2%	1.99 [1.15, 3.44]
Neiswanger 1995	30	104	4	60	0.1%	0.4%	5.68 [1.89, 17.04]
Goldman 1997	322	552	196	322	3.3%	2.5%	0.90 [0.68, 1.19]
Gelernter 1999	55	320	48	272	1.4%	1.6%	0.97 [0.63, 1.48]
Konishi 2004	180	400	219	502	3.4%	2.7%	1.06 [0.81, 1.38]
Sakai 2007	131	478	199	774	3.6%	2.7%	1.09 [0.84, 1.41]
**Total (fixed effect, 95% CI)**		**2878**		**3035**	**15.2%**	--	**1.24 [1.10, 1.40]**
**Total (random effects, 95% CI)**					--	**16.7%**	**1.50 [1.15, 1.95]**
Heterogeneity: $Tau^2 = 0.16$; $Chi^2 = 47.72$, df = 14 (P < 0.01); $I^2 = 71\%$							
Test for overall effect (fixed effect): Z = 3.56 (P < 0.01)							
Test for overall effect (random effects): Z = 2.98 (P < 0.01)							
**region = Europe**							
Amadeo 1993	24	98	7	86	0.2%	0.5%	3.66 [1.49, 9.00]
Geijer 1994	26	148	34	162	0.9%	1.1%	0.80 [0.45, 1.42]
Sander 1995	99	540	43	226	1.6%	1.8%	0.96 [0.64, 1.42]
Finckh 1996	112	624	42	262	1.6%	1.8%	1.15 [0.78, 1.69]
Heinz 1996	37	194	43	226	1.0%	1.4%	1.00 [0.62, 1.63]
Hietala 1997	31	140	11	100	0.3%	0.7%	2.30 [1.09, 4.84]
Ovchinnikov 1999	33	84	31	152	0.4%	1.0%	2.53 [1.40, 4.55]
Sander 1999	110	620	66	392	2.1%	2.2%	1.07 [0.76, 1.49]
Amadeo 2000	49	138	41	114	0.9%	1.3%	0.98 [0.58, 1.64]
Gorwood_a 2000	47	226	21	138	0.7%	1.1%	1.46 [0.83, 2.57]
Gorwood_b 2000	16	72	30	96	0.6%	0.8%	0.63 [0.31, 1.27]
Samochowiec 2000	104	584	61	384	1.9%	2.1%	1.15 [0.81, 1.62]
Anghelescu 2001	101	486	38	196	1.4%	1.7%	1.09 [0.72, 1.65]
Pastorelli 2001	19	120	17	128	0.4%	0.8%	1.23 [0.61, 2.49]
Limosin 2002	61	240	43	214	1.1%	1.6%	1.36 [0.87, 2.11]
Berggren 2006	159	714	296	1684	4.4%	3.1%	1.34 [1.08, 1.67]
Samochowiec 2008	43	244	54	300	1.3%	1.6%	0.97 [0.63, 1.52]
Kraschewski 2009	134	720	129	736	3.3%	2.6%	1.08 [0.82, 1.41]
Kovanen 2010	233	1024	196	1022	4.9%	3.1%	1.24 [1.00, 1.54]
Kasiakogia-Worlley 2011	395	2012	389	1910	10.3%	3.6%	0.96 [0.82, 1.12]
Landgren 2011	33	168	14	64	0.5%	0.8%	0.87 [0.43, 1.77]
Mignini 2012	118	550	89	546	2.3%	2.4%	1.40 [1.03, 1.90]
Schellekens 2012	39	220	31	198	0.9%	1.3%	1.16 [0.69, 1.95]
Jasiewicz 2014	74	338	59	314	1.5%	1.9%	1.21 [0.83, 1.78]
**Total (fixed effect, 95% CI)**		**10304**		**9650**	**44.7%**	--	**1.14 [1.06, 1.23]**
**Total (random effects, 95% CI)**					--	**40.1%**	**1.16 [1.05, 1.28]**
Heterogeneity: $Tau^2 = 0.02$; $Chi^2 = 34.86$, df = 23 (P = 0.05); $I^2 = 34\%$							
Test for overall effect (fixed effect): Z = 3.64 (P < 0.01)							
Test for overall effect (random effects): Z = 3.01 (P < 0.01)							
**region = Asia**							
Arinami 1993	63	156	23	70	0.6%	1.0%	1.38 [0.77, 2.50]
Chen 1996	129	316	32	82	1.0%	1.3%	1.08 [0.66, 1.77]
Lu 1996	52	122	54	130	1.0%	1.3%	1.05 [0.63, 1.72]
Chen 1997	166	406	163	426	3.0%	2.6%	1.12 [0.85, 1.47]
Kono 1997	78	200	69	186	1.4%	1.7%	1.08 [0.72, 1.64]
Lee 1997	123	256	66	170	1.3%	1.8%	1.46 [0.98, 2.16]
Ishiguro 1998	179	418	106	304	2.3%	2.4%	1.40 [1.03, 1.90]
Lu 2001	98	194	66	170	1.1%	1.7%	1.61 [1.06, 2.44]
Shaikh 2001	42	100	45	106	0.8%	1.2%	0.98 [0.56, 1.71]
Huang 2007	191	452	141	402	2.8%	2.6%	1.35 [1.03, 1.79]
Wang 2007	68	146	132	316	1.4%	1.8%	1.22 [0.82, 1.80]
Joe 2008	425	1058	218	546	5.5%	3.1%	1.01 [0.82, 1.25]
Bhaskar 2010	75	162	109	230	1.6%	1.8%	0.96 [0.64, 1.43]
Prasad 2010	48	180	26	120	0.7%	1.2%	1.31 [0.76, 2.27]
Lu 2012	115	266	313	816	2.8%	2.5%	1.22 [0.92, 1.62]
Hu 2013	85	202	228	656	2.0%	2.2%	1.36 [0.99, 1.88]
Suraj Singh 2013	100	258	171	572	2.1%	2.3%	1.48 [1.09, 2.02]
**Total (fixed effect, 95% CI)**		**4892**		**5302**	**31.5%**	--	**1.22 [1.12, 1.33]**
**Total (random effects, 95% CI)**					--	**32.5%**	**1.22 [1.12, 1.33]**
Heterogeneity: $Tau^2 = 0$; $Chi^2 = 12.43$, df = 16 (P = 0.71); $I^2 = 0\%$							
Test for overall effect (fixed effect): Z = 4.60 (P < 0.01)							
Test for overall effect (random effects): Z = 4.60 (P < 0.01)							
**region = Australia**							
Lawford 1997	82	402	27	94	1.1%	1.3%	0.64 [0.38, 1.06]
Foley 2004	53	174	47	208	1.0%	1.5%	1.50 [0.95, 2.37]
**Total (fixed effect, 95% CI)**		**576**		**302**	**2.1%**	--	**1.03 [0.73, 1.46]**
**Total (random effects, 95% CI)**					--	**2.8%**	**0.98 [0.42, 2.28]**
Heterogeneity: $Tau^2 = 0.31$; $Chi^2 = 6.05$, df = 1 (P = 0.01); $I^2 = 83\%$							
Test for overall effect (fixed effect): Z = 0.19 (P = 0.85)							
Test for overall effect (random effects): Z = -0.04 (P = 0.97)							
**region = South America**							
Bau 2000	62	230	48	228	1.1%	1.6%	1.38 [0.90, 2.13]
Freire 2006	55	200	98	464	1.4%	1.9%	1.42 [0.97, 2.08]
Vasconcelos 2015	89	226	72	228	1.4%	1.8%	1.41 [0.96, 2.07]
**Total (fixed effect, 95% CI)**		**656**		**920**	**3.9%**	--	**1.40 [1.12, 1.77]**
**Total (random effects, 95% CI)**					--	**5.3%**	**1.40 [1.12, 1.77]**
Heterogeneity: $Tau^2 = 0$; $Chi^2 = 0.01$, df = 2 (P = 1.00); $I^2 = 0\%$							
Test for overall effect (fixed effect): Z = 2.89 (P < 0.01)							
Test for overall effect (random effects): Z = 2.89 (P < 0.01)							
**region = Central America**							
Panduro 2017	335	624	126	284	2.6%	2.5%	1.45 [1.10, 1.93]
**Total (fixed effect, 95% CI)**		**624**		**284**	**2.6%**	--	**1.45 [1.10, 1.93]**
**Total (random effects, 95% CI)**					--	**2.5%**	**1.45 [1.10, 1.93]**
Heterogeneity: not applicable							
Test for overall effect (fixed effect): Z = 2.60 (P < 0.01)							
Test for overall effect (random effects): Z = 2.60 (P < 0.01)							
**Total (fixed effect, 95% CI)**		**19930**		**19493**	**100.0%**	--	**1.20 [1.14, 1.26]**
**Total (random effects, 95% CI)**					--	**100.0%**	**1.23 [1.14, 1.31]**
Heterogeneity: $Tau^2 = 0.03$; $Chi^2 = 107.20$, df = 61 (P < 0.01); $I^2 = 43\%$							
Residual heterogeneity: Tau^2 = NA; $Chi^2 = 101.08$, df = 56 (P < 0.01); $I^2 = 45\%$							
Test for overall effect (fixed effect): Z = 7.46 (P < 0.01)							
Test for overall effect (random effects): Z = 5.79 (P < 0.01)							
Test for subgroup differences (fixed effect): $Chi^2 = 6.51$, df = 5 (P = 0.26)							
Test for subgroup differences (random effects): $Chi^2 = 6.26$, df = 5 (P = 0.28)							

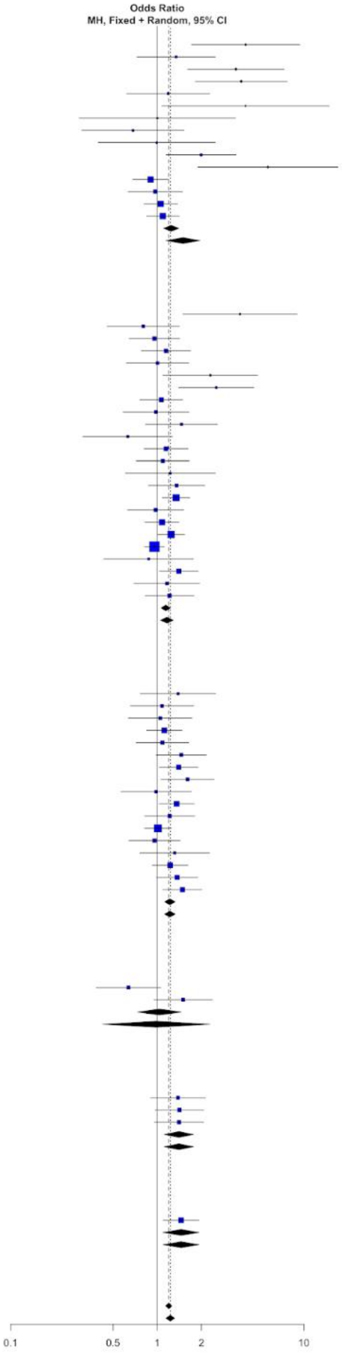

eFigure 1. Meta-analysis of *DRD2* rs1800497/AUD Studies Stratified by Region.

Studies were performed in five world regions, Europe, North America, South America, Central

© 2019 Jung Y et al. *JAMA Network Open.*

America, Asia and Australia as shown. *Black bars and blue squares* depict 95% confidence intervals (CIs) and odds ratios (ORs) in each study. Pooled effect sizes were estimated under fixed effects and random effects models.

© 2019 Jung Y et al. *JAMA Network Open.*

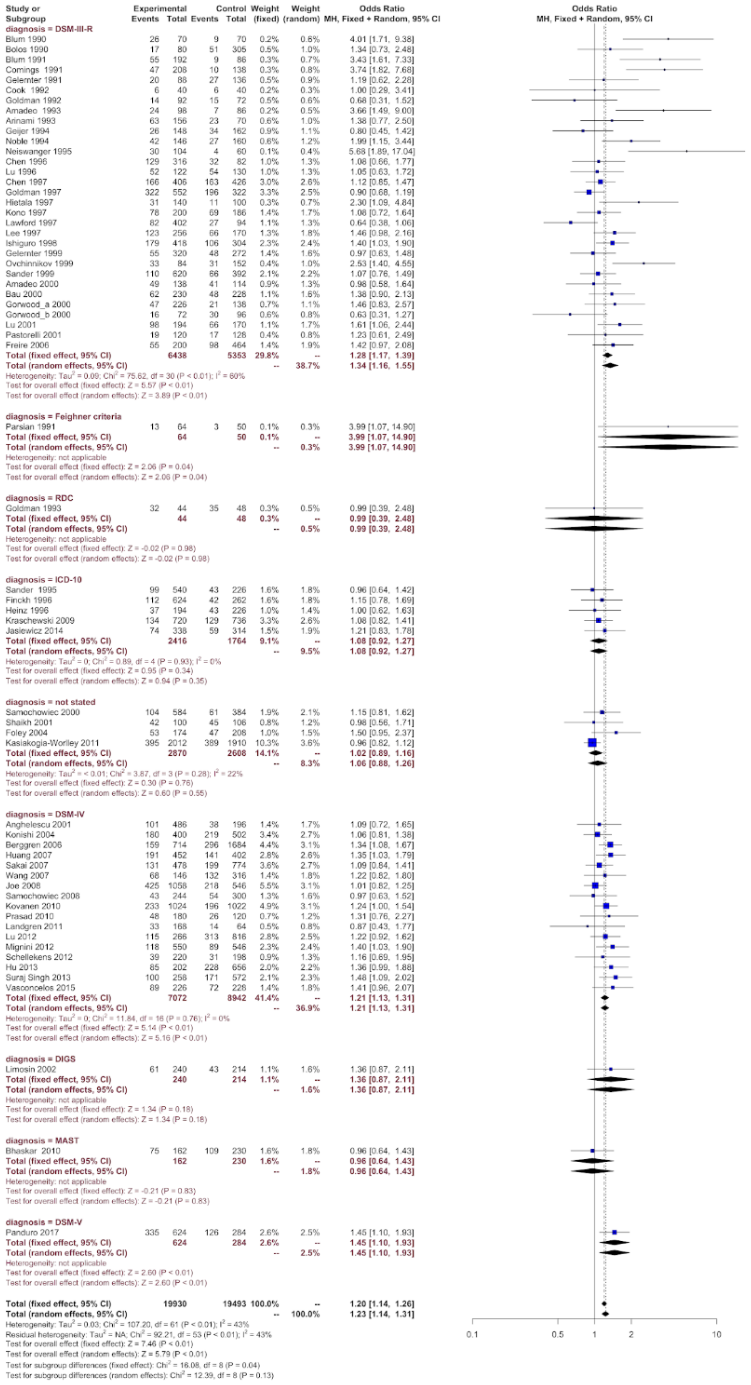

eFigure 2. **Meta-analysis of *DRD2* rs1800497/AUD Studies Stratified by Diagnostic Criteria.**

Studies were performed using nine different types of diagnostic criteria as shown. *Black bars*

© 2019 Jung Y et al. *JAMA Network Open.*

and blue squares depict 95% confidence intervals (CIs) and odds ratios (ORs) in each study. Pooled effect sizes were estimated under fixed effects and random effects models.

© 2019 Jung Y et al. *JAMA Network Open.*

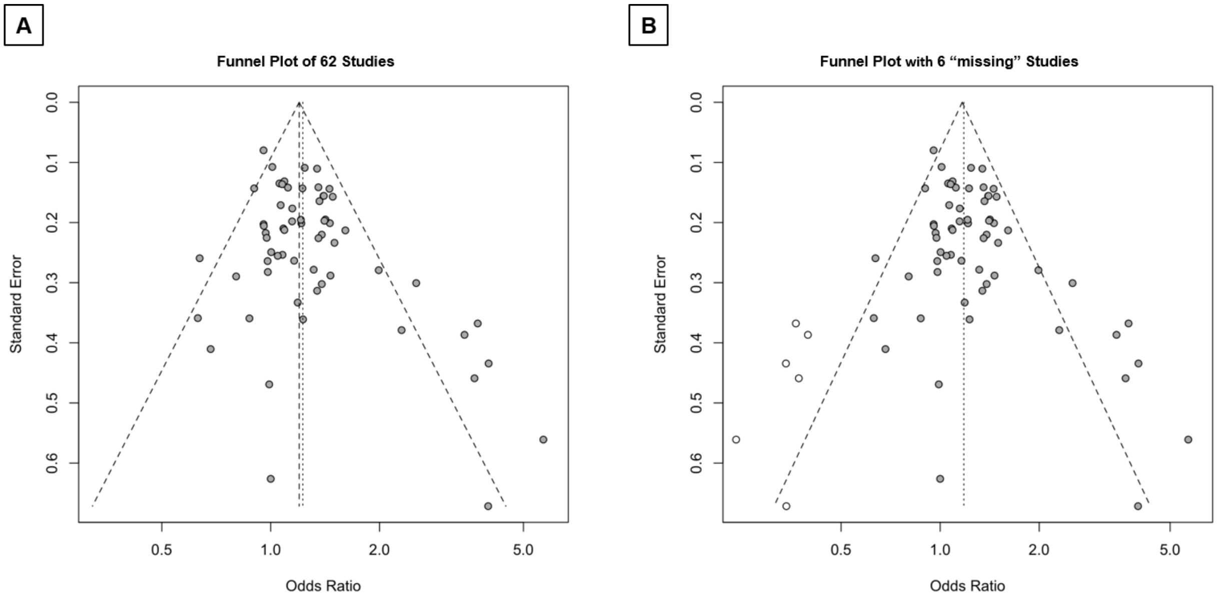

eFigure 3. Publication Bias

(A) Publication bias in *DRD2* (rs1800497)/AUD association studies and (B) prediction of six "missing" studies via the trim and fill model. Solid line at OR=1.23 is the meta-analytic OR derived from 62 published studies. Publication bias was estimated by Begg's rank correlation (p-0.106) and Egger's regression test (p=0.004).

© 2019 Jung Y et al. *JAMA Network Open.*

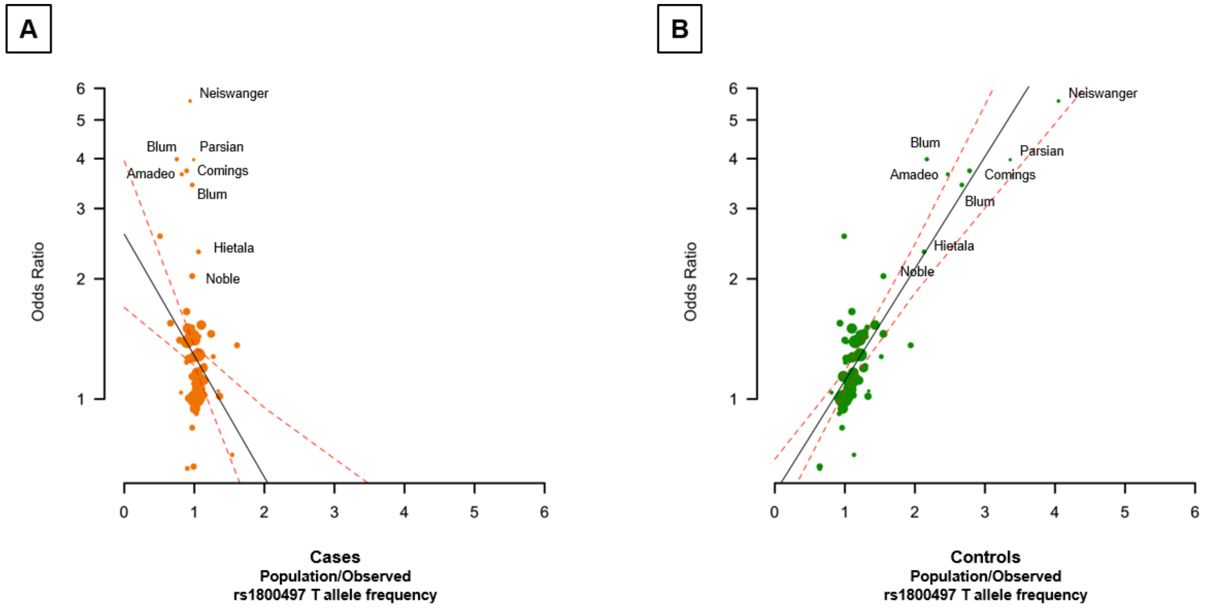

eFigure 4. **Meta-Regression Plots**

Meta-regression plots of (A) Case allele frequency ratio (B) Control allele frequency ratio (C) Case allele frequency ratio in North American studies (D) Control allele frequency ratio in North American studies. In each case, allele frequency is compared to population allele frequency in ExAC to detect allele frequency deviation. For comparison to 1000 Genomes allele frequencies, see Figure 4. Diameters of circles are proportional to study population size. Solid line represents the meta-regression slope of relationship of odds ratio to allele frequency deviation. (A) z= -3.36, p<0.0008 (B) z=7.76, p<0.0001.

© 2019 Jung Y et al. *JAMA Network Open.*

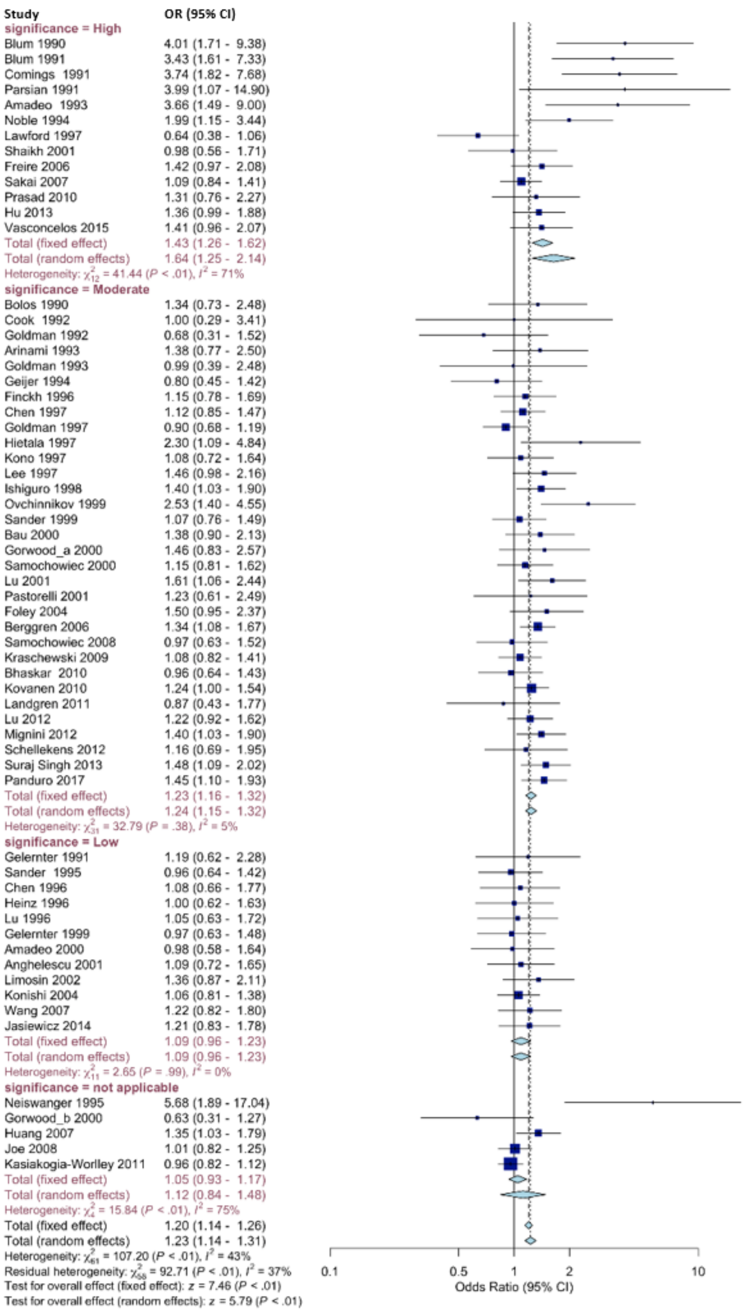

eFigure 5. **Meta-analysis of rs1800497 Association With AUD Stratified by Deviation Significance**

Deviation significance was reported in each study (high, moderate and low). *Black bars and blue squares* depict 95% confidence intervals (CIs) and odds ratios (ORs) in each study. Pooled

© 2019 Jung Y et al. *JAMA Network Open.*

effect sizes were estimated under fixed effects and random effects models.

© 2019 Jung Y et al. *JAMA Network Open.*

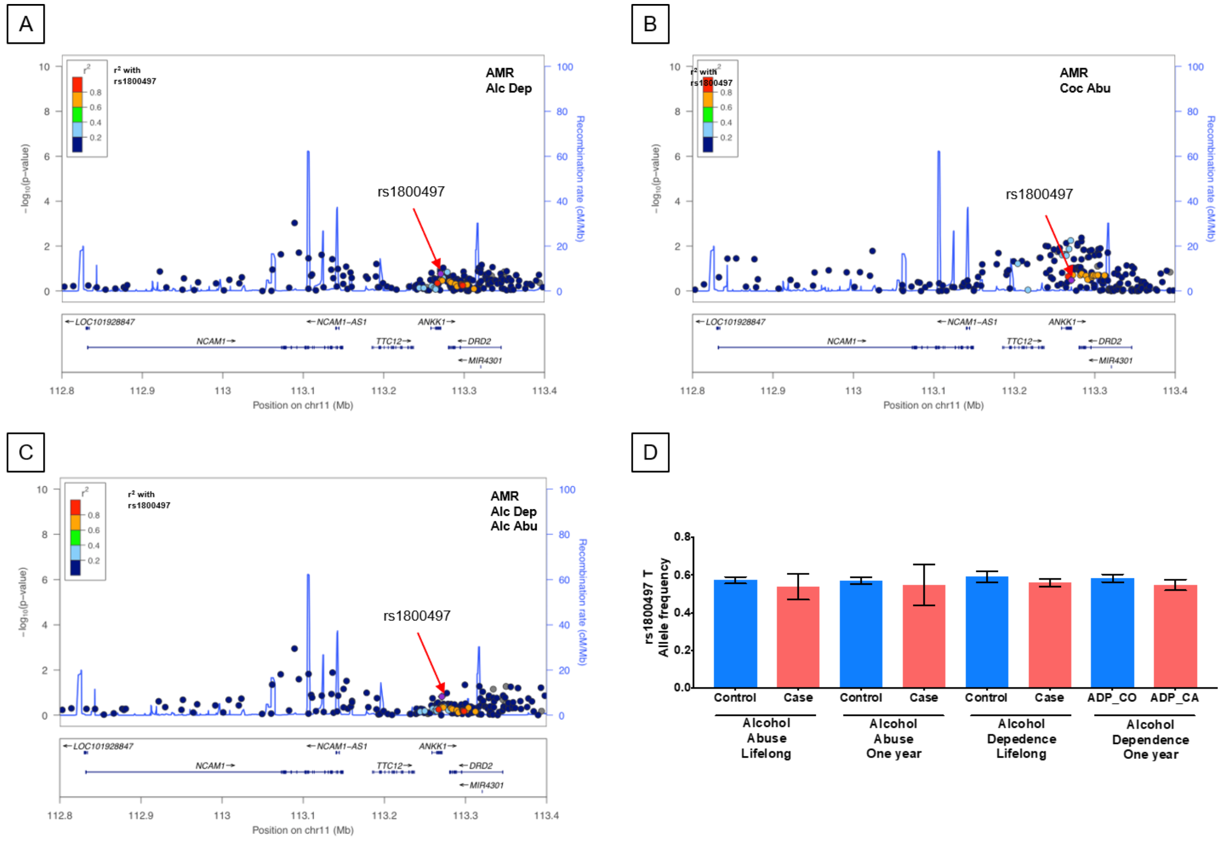

eFigure 6. Association of SNPs in the *DRD2* Region (Native Americans)

Regional association plot of rs1800497 and a total of 201 SNPs in the *DRD2* region to (A) Alcohol dependence, (B) Cocaine abuse, and (C) Alcohol dependence and abuse, in Native Americans (N=501). SNPs are color coded according to linkage disequilibrium (LD) with rs1800497 (purple dot with red arrow) on a scale of r^2 0 to 1. Estimated recombination rates reflect local LD structure in the 600 kb buffer around rs1800497 in this Native American population. rs1800497 allele frequencies (D) did not differ between cases and controls.

© 2019 Jung Y et al. *JAMA Network Open.*

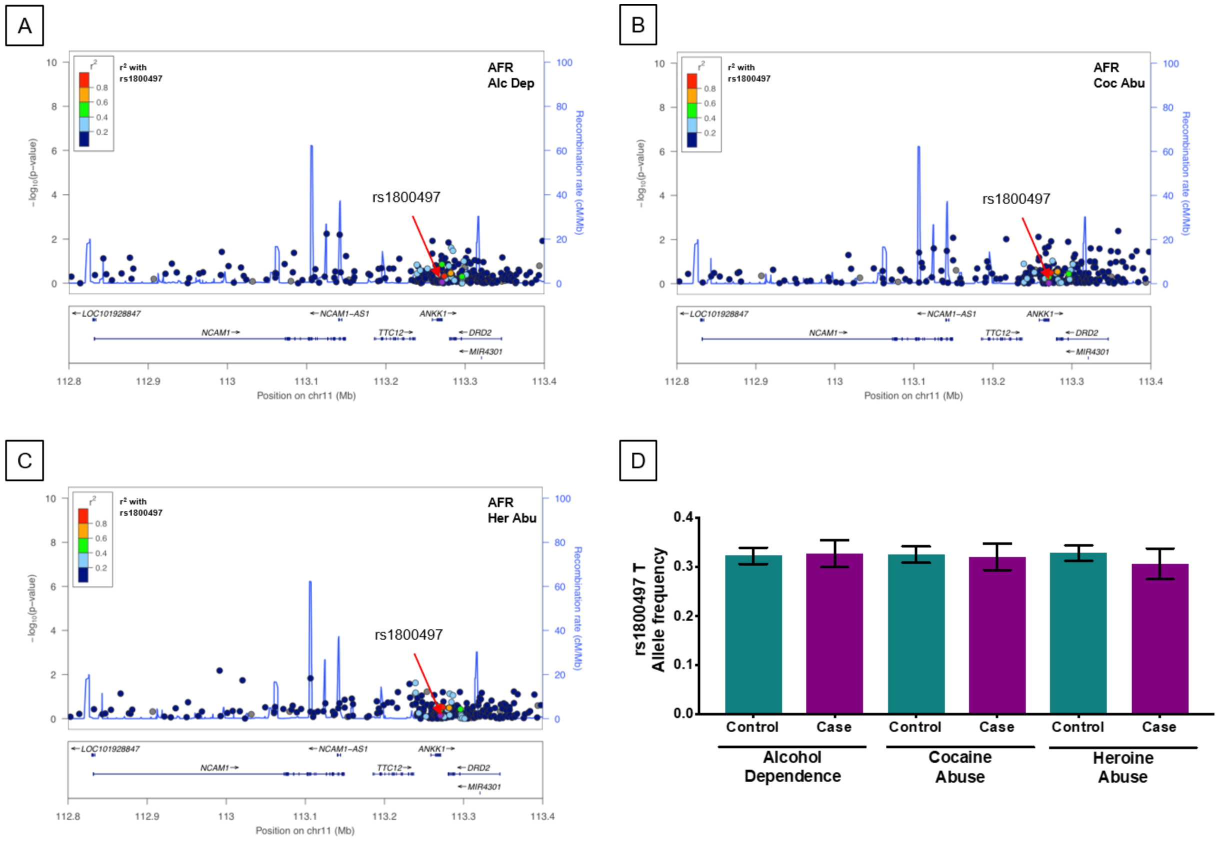

eFigure 7. Association of SNPs in the *DRD2* Region (African Americans)

Regional association plot of rs1800497 and a total of 260 SNPs in the *DRD2* region to (A) Alcohol dependence, (B) Cocaine abuse, and (C) Heroin abuse, in African Americans (N=583). SNPs are color coded according to linkage disequilibrium (LD) with rs1800497 (purple dot with red arrow) on a scale of r^2 0 to 1. Estimated recombination rates reflect local LD structure in the 600 kb buffer around rs1800497 in this African American population. rs1800497 allele frequencies (D) did not differ between cases and controls.

© 2019 Jung Y et al. *JAMA Network Open.*

eTable. Deviations of Case and Control Allele Frequencies in *DRD2* (rs1800497)/AUD Association Studies (ExAC Allele Frequencies)

			Alcohol Use Disorder		Control		
			Significance of deviation		Significance of deviation		
Studies	N	Diagnostic Method	E/O	P-value	E/O	P-value	Study OR (95% CI)
Blum 1990	140	DSM-III-R	0.89	0.143	2.57	0.017	4.01 (1.71-9.38)
Bolos 1990	385	DSM-III-R	0.89	0.825	0.97	0.872	1.34 (0.73-2.48)
Blum 1991	278	DSM-III-R	1.15	0.845	3.15	0.002	3.43 (1.61-7.33)
Comings 1991	346	DSM-III-R	0.84	0.430	2.62	0.001	3.74 (1.82-7.68)
Gelernter 1991	224	DSM-III-R	0.84	0.589	0.96	0.941	1.19 (0.62-2.28)
Parsian 1991	114	Feighner criteria	0.94	0.975	3.17	0.026	3.99 (1.07-14.90)
Cook 1992	80	DSM-III-R	1.27	0.469	1.27	0.469	1.00 (0.29-3.41)
Goldman 1992	164	DSM-III-R	1.12	0.103	0.82	0.800	0.68 (0.31-1.52)
Amadeo 1993	184	DSM-III-R	0.78	0.337	2.34	0.013	3.66 (1.49-9.00)
Arinami 1993	226	DSM-III-R	1.02	0.658	1.25	0.208	1.38 (0.77-2.50)
Goldman 1993	92	RDC	0.81	0.232	0.81	0.206	0.99 (1.15-3.44)
Geijer 1994	310	DSM-III-R	1.08	0.486	0.91	0.810	0.80 (0.45-1.42)
Noble 1994	306	DSM-III-R	1.15	0.843	1.96	0.008	1.99 (1.15-3.44)
Neiswanger 1995	164	DSM-III-R	0.94	NA	4.05	NA	5.68 (1.89-17.04)
Sander 1995	766	ICD-10	1.04	0.350	1.00	0.709	0.96 (0.64-1.42)
Chen 1996	398	DSM-III-R	1.00	0.610	1.05	0.611	1.08 (0.66-1.77)
Finckh 1996	886	ICD-10	1.06	0.223	1.19	0.138	1.15 (0.78-1.69)
Heinz 1996	420	ICD-10	1.00	0.740	1.00	0.709	1.00 (0.62-1.63)
Lu 1996	252	DSM-III-R	0.91	0.990	0.99	0.839	1.05 (0.63-1.72)
Chen 1997	832	DSM-III-R	1.00	0.576	1.07	0.161	1.12 (0.85-1.47)
Goldman 1997	874	DSM-III-R	1.01	0.862	0.97	0.645	0.90 (0.68-1.19)
Hietala 1997	240	DSM-III-R	0.86	0.751	1.73	0.010	2.30 (1.09-4.84)
Kono 1997	386	DSM-III-R	1.05	0.423	1.11	0.242	1.08 (0.72-1.64)
Lawford 1997	496	DSM-III-R	0.93	0.908	0.66	0.020	0.64 (0.38-1.06)
Lee 1997	426	DSM-III-R	0.85	0.190	1.06	0.439	1.46 (0.98-2.16)
Ishiguro 1998	722	DSM-III-R	0.96	0.969	1.18	0.037	1.40 (1.03-1.90)
Gelernter 1999	592	DSM-III-R	0.99	0.021	0.96	0.048	0.97 (0.63-1.48)
Ovchinnikov 1999	236	DSM-III-R	0.48	0.000	0.93	0.944	2.53 (1.40-4.55)
Sander 1999	1012	DSM-III-R	1.07	0.176	1.13	0.145	1.07 (0.76-1.49)
Amadeo 2000	252	DSM-III-R	0.99	0.920	0.97	0.862	0.98 (0.58-1.64)
Bau 2000	458	DSM-III-R	1.00	0.990	1.28	0.084	1.38 (0.90-2.13)
Gorwood_a 2000	364	DSM-III-R	0.91	0.826	1.25	0.198	1.46 (0.83-2.57)
Gorwood_b 2000	168	DSM-III-R	0.9	NA	0.64	NA	0.63 (0.31-1.27)
Samochowiec 2000	968	not stated	1.07	0.209	1.20	0.063	1.15 (0.81-1.62)
Anghelescu 2001	682	DSM-IV	0.91	0.753	0.98	0.814	1.09 (0.72-1.65)
Lu 2001	364	DSM-III-R	0.81	0.243	1.06	0.439	1.61 (1.06-2.44)
Pastorelli 2001	248	DSM-III-R	1.20	0.293	1.43	0.084	1.23 (0.61-2.49)
Shaikh 2001	206	not stated	0.98	1.000	0.97	0.943	0.98 (0.56-1.71)
Limosin 2002	454	DIGS	0.75	0.069	0.95	0.988	1.36 (0.87-2.11)
Foley 2004	382	not stated	0.62	0.002	0.87	0.640	1.50 (0.95-2.37)
Konishi 2004	902	DSM-IV	0.98	0.262	1.01	0.090	1.06 (0.81-1.38)
Berggren 2006	2398	DSM-IV	0.85	0.205	1.08	0.019	1.34 (1.08-1.67)
Freire 2006	664	DSM-III-R	0.98	0.892	1.28	0.015	1.42 (0.97-2.08)
Huang 2007	854	DSM-IV	1.01	NA	1.22	NA	1.35 (1.03-1.79)
Sakai 2007	1252	DSM-IV	1.20	0.838	1.23	0.822	1.09 (0.84-1.41)
Wang 2007	462	DSM-IV	0.88	0.474	0.98	0.801	1.22 (0.82-1.80)
Joe 2008	1604	DSM-IV	1.06	NA	1.07	NA	1.01 (0.82-1.25)
Samochowiec 2008	544	DSM-IV	1.06	0.381	1.06	0.409	0.97 (0.63-1.52)
Kraschewski 2009	1456	ICD-10	1.02	0.361	1.09	0.114	1.08 (0.82-1.41)
Bhaskar 2010	392	MAST	0.89	0.484	0.87	0.276	0.96 (0.64-1.43)
Kovanen 2010	2046	DSM-IV	0.75	0.343	0.89	0.005	1.24 (1.00-1.54)
Prasad 2010	300	DSM-IV	1.55	0.001	1.86	0.093	1.31 (0.76-2.27)
Kasiakogia-Worlley 2011	3922	not stated	1.02	NA	0.98	NA	0.96 (0.82-1.12)
Landgren 2011	232	DSM-IV	0.97	0.886	0.87	0.757	0.87 (0.43-1.77)
Lu 2012	1082	DSM-IV	0.95	0.894	1.07	0.058	1.22 (0.92-1.62)
Mignini 2012	1096	DSM-IV	0.89	0.492	1.17	0.046	1.40 (1.03-1.90)
Schellekens 2012	418	DSM-IV	1.07	0.425	1.22	0.160	1.16 (0.69-1.95)
Hu 2013	858	DSM-IV	0.97	0.893	1.18	0.002	1.36 (0.99-1.88)
Suraj Singh 2013	830	DSM-IV	1.06	0.333	1.37	0.000	1.48 (1.09-2.02)
Jasiewicz 2014	652	ICD-10	0.87	0.473	1.01	0.594	1.21 (0.83-1.78)
Vasconcelos 2015	454	DSM-IV	1.12	0.040	1.39	0.000	1.41 (0.96-2.07)
Panduro 2017	908	DSM-V	0.89	0.089	1.08	0.273	1.45 (1.10-1.93)

© 2019 Jung Y et al. *JAMA Network Open.*
